# Late recurrence of Kikuchi–Fujimoto disease nine years after initial diagnosis: a case-based review

**DOI:** 10.1007/s10067-026-08134-7

**Published:** 2026-04-30

**Authors:** Arian Akhondi, Valerie Anne-Sophie Faber, Adam Hawkins, David Ray Chen

**Affiliations:** 1 Division of Hematology Oncology, Department of Medicine, Perelman School of Medicine, University of Pennsylvania, Philadelphia, PA USA; 2 Division of Internal Medicine, University Hospital of Vienna, Vienna, Austria; 3 Division of Rheumatology, Department of Medicine, Perelman School of Medicine, University of Pennsylvania, Philadelphia, PA USA

**Keywords:** Autoimmune disease, Kikuchi-Fujimoto disease (KFD), Necrotizing lymphadenitis

## Abstract

Kikuchi–Fujimoto disease (KFD), also known as histiocytic necrotizing lymphadenitis, is a rare, benign, and self-limited inflammatory lymphadenitis. It predominantly affects young adults and typically presents with tender cervical lymphadenopathy, low-grade fever, and constitutional symptoms such as malaise, night sweats, and weight loss. Though initially recognized in Asian populations, cases are now reported worldwide. The etiology remains uncertain, but KFD has recognized overlap with autoimmune diseases. Systemic lupus erythematosus (SLE) and Sjögren’s disease (SjD) account for most coexisting disorders. We present the case of a woman in her early forties with recurrent cyclical fevers and cervical lymphadenopathy. She had biopsy-proven KFD but showed relapse after a nine-year asymptomatic period after her first manifestation. A case-based review about KFD recurrence was conducted using PubMed/MEDLINE databases following CaBArET guidelines and identified a total of 21 reports for review of the clinical manifestations and treatment approaches of recurrent KFD. Our findings suggest that although KFD is typically self-limited, recurrence may be associated with autoimmune features in selected cases, emphasizing the need for renewed diagnostic vigilance even years after apparent resolution. Although treatment responses in recurrent Kikuchi–Fujimoto disease are heterogeneous, literature evidence and our case suggest that corticosteroids remain first-line, while agents such as hydroxychloroquine or anakinra may offer benefit in steroid-dependent or relapsing cases. However, these remain individualized interventions rather than standardized protocols, and controlled evidence is lacking.

**Table:** 

**Key Points** • *Kikuchi–Fujimoto disease, although typically self-limited, can relapse after unusually long latency, highlighting the need for continued clinical awareness.* • *Recurrent presentations may mimic infectious or malignant conditions, often leading to extensive diagnostic work-ups.* • *Reactive histologic findings at recurrence do not exclude KFD and should be interpreted in the context of prior confirmed disease and clinical evolution.*

**Supplementary Information:**

The online version contains supplementary material available at 10.1007/s10067-026-08134-7.

## Introduction

Kikuchi–Fujimoto disease (KFD), also known as histiocytic necrotizing lymphadenitis, is an uncommon, self-limited inflammatory disorder first described independently by Kikuchi and Fujimoto in 1972 (1,2). It typically affects young women and presents with tender cervical lymphadenopathy, fever, and constitutional symptoms such as malaise and night sweats (3). Although the course is usually benign, its clinical and histopathological features often mimic lymphoma or systemic lupus erythematosus (SLE), posing significant diagnostic challenges (4,5). The etiology of KFD remains incompletely understood. Infectious triggers—most notably Epstein–Barr virus, cytomegalovirus, and parvovirus B19—have been implicated, yet no causative pathogen has been confirmed (6,7). Alternatively, autoimmune mechanisms have gained support from associations with antinuclear antibodies, autoimmune thyroiditis, SLE, and Sjögren’s disease (SjD) (6). Genetic susceptibility has also been suggested through HLA-DPA1/DPB1 polymorphisms, further linking KFD to immune dysregulation (8).

While KFD generally follows a monophasic course resolving within weeks to months, relapse and atypical presentations are increasingly recognized (9). Neurological involvement, most frequently aseptic meningitis, and cutaneous manifestations such as leukocytoclastic vasculitis or lupus-like eruptions have been reported (3,10,11).

We present a case of biopsy-proven late recurrent Kikuchi–Fujimoto disease occurring nine years after initial remission followed by a case-based review of the literature to investigate recurrence patterns, associated conditions, and optimal management strategies.

## Case-based review

A woman in her early thirties, previously healthy except for one caesarean delivery, first presented in 2016 with cyclical fevers recurring every few weeks for two months, each episode lasting 7 to 10 days with temperatures up to 40 °C. She reported malaise and myalgias without respiratory, urinary, or gastrointestinal symptoms. She had been hospitalized twice elsewhere for pancytopenia; cultures, imaging, and bone marrow biopsy were unrevealing.

On arrival, she was febrile and diaphoretic but alert.

Her vitals on arrival showed a slightly elevated blood pressure of 134/81 mmHg, slight tachycardia with 126 beats per minute, a febrile oral temperature of 39.2 °C, and a respiratory rate of 18 with oxygen saturation of 100% on room air.

Her clinical exam showed a palpable, tender left cervical and right supraclavicular lymphadenopathy, however no rash, arthritis, or hepatosplenomegaly. Given recurrent fever and cytopenias, she was admitted for further evaluation. Her laboratory values are displayed in Table 1.

**Table 1:** Selected laboratory values on admission in 2016

Test	Result	Reference
WBC	1.2 × 10⁹/L	↓
Hb	10.2 g/dL	↓
Platelets	159 × 10⁹/L	N
Ferritin	423 ng/mL	↑
CRP	56.3 mg/L	↑
ESR	47 mm/h	↑
LDH	312 U/L	↑
ANA/RF/SSA/SSB	Negative	O
Complements C3/C4	Normal	O
HIV, HBV, HCV, viral panel	Negative	O

Imaging was performed given the clinical findings of enlarged lymph nodes. Computed tomography of the neck revealed bilateral level 2 and level 5 cervical lymphadenopathy up to 1.6 cm with preserved architecture. This finding is consistent with systemic inflammatory disease. The chest showed scattered sub-5 mm pulmonary nodules with mild symmetric axillary/supraclavicular lymphadenopathy and no mediastinal or hilar nodes. An additional transthoracic echocardiography showed no significant abnormalities with a left ventricular ejection fraction of 55–60%. Given the suspicion of hematological malignancy, a bone marrow biopsy was performed. The histopathology showed normocellular (~ 60%) bone marrow with trilineage hematopoiesis, megakaryocytic hyperplasia, and a minute nonnecrotizing granuloma. While a lung biopsy showed unremarkable findings, an excisional cervical lymph node biopsy showed features of necrotizing histiocytic lymphadenitis with abundant MPO-positive histiocytes and CD8 T-cells. Neutrophils and eosinophils were absent and could not be appreciated. Rheumatology suggested first manifestation of Kikuchi–Fujimoto disease. Lupus lymphadenitis and peripheral T-cell lymphoma were considered less likely after the histopathology of the lymph node biopsy came back. Molecular studies excluded lymphoma.

After nine years of remission, the patient—now 42 years old—presented in October 2025 with recurrent cyclical fevers, arthralgias, rash, and neck stiffness. She denied respiratory or abdominal symptoms. Her laboratory values on re-admission are as displayed in Table 2.

**Table 2:** Selected laboratory values on re-admission 2025

Test	Result	Reference
WBC	2.5 × 10⁹/L	↓
Platelets	149 × 10⁹/L	↓
Ferritin	725 ng/mL	↑
CRP	7.6 mg/L	↑
ESR	36 mm/h	< 20 mm/h
ANA, anti-dsDNA, RF, ANCA	Negative	O
Complements C3/C4	normal	O
SSA	138.4 U/mL	O
SSB	22.6 U/mL	O

**Fig. 1:**
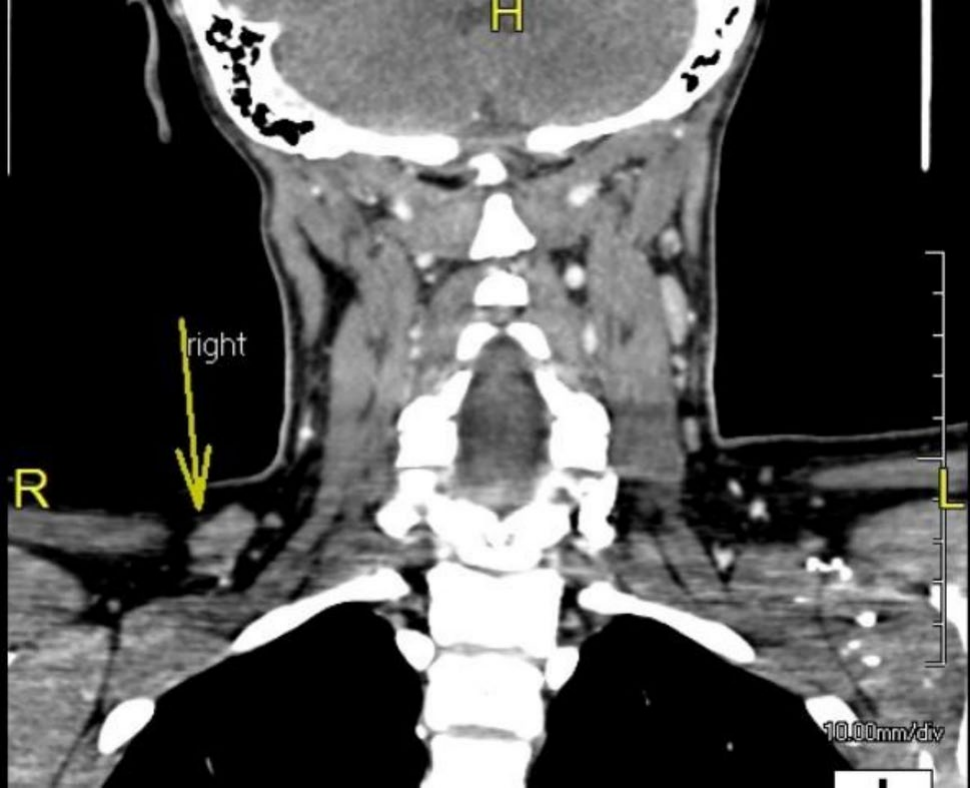
Radiographical finding of right enlarged cervical lymph node during 2025 hospitalization

Extensive testing for bacterial, viral, and tick-borne pathogens was performed. HIV, COVID-19, influenza, EBV IgM, CMV, parvovirus B19, histoplasma, malaria, blood parasites, syphilis (Rapid Plasma Reagin), Lyme disease, Anaplasma, Ehrlichia, Babesia, and Rickettsia rickettsii all came back negative. An empiric doxycycline (10 Oct 2025) was started on admission to cover possible tick-borne infection but was discontinued after 2.5 days owing to lack of improvement and negative results. The patient had also been started on ampicillin, ceftriaxone, and acyclovir for possible meningitis that were also stopped once infection was excluded. A hematological and oncological evaluation was requested for suspicion of malignancy. However, a peripheral flow cytometry and bone marrow morphology excluded malignancy. CT chest/abdomen/pelvis revealed no lymphoproliferative disease, while an axillary lymph node seemed enlarged (Fig. 1).

**Fig. 2:**
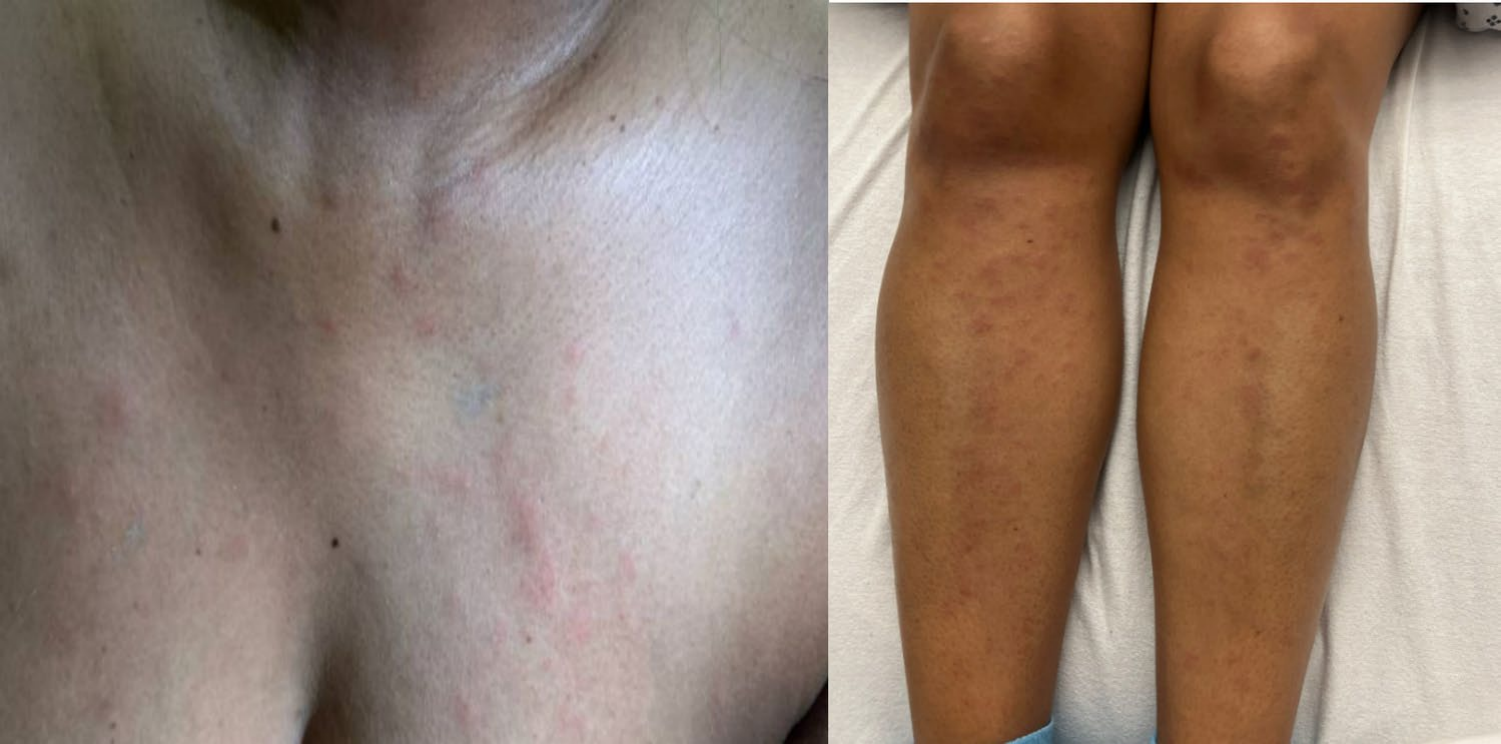
Findings on clinical examination on 2025 presentation

On clinical examination, the patient showed rashes on her chest and lower extremities (Fig. 2). The department for dermatology suggested biopsy. The skin biopsy from her right lower thigh showed slight epidermal thickening with a dense superficial and deep perivascular lymphocytic infiltrate. Focal vessel wall damage suggestive of lymphocytic vasculitis was described and periodic acid-Schiff stain with diastase and immunofluorescence both were negative. Findings were considered consistent with a possible cutaneous manifestation of KFD.

**Fig. 3:**
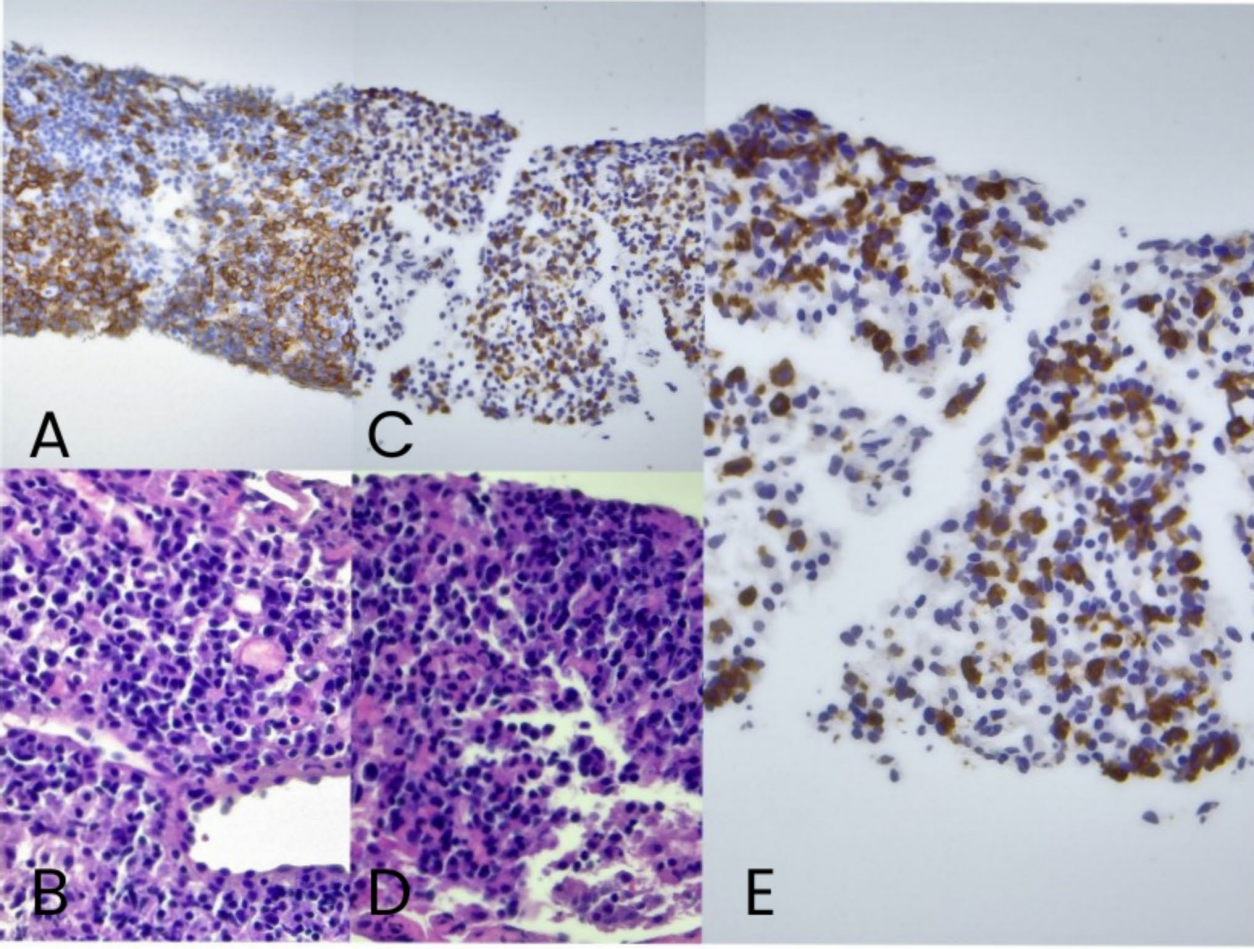
Excisional lymph node biopsy showing an interfollicular polyclonal plasma cell population without light chain restriction during the 2025 admission. **A** CD138 immunostaining highlights numerous plasma cells expanding the interfollicular regions (× 200). **B** H&E (× 400) shows interfollicular plasma cell infiltrate with preserved nodal architecture. **C** Kappa in situ hybridization demonstrates kappa-expressing plasma cells (× 200). **D** H&E (× 400) from a second area shows similar interfollicular plasma cell infiltrate with preserved architecture. **E** Lambda in situ hybridization demonstrates lambda-expressing plasma cells (× 400)

A left axillary lymph node core biopsy showed a reactive lymph node with polytypic κ/λ plasma cells by in situ hybridization, no lymphoma, granulomas, or necrosis. It was interpreted by an expert hematopathologist as reactive lymphadenitis in an autoimmune/inflammatory context (Fig. 3). Although the 2025 biopsy did not demonstrate classical necrosis, recurrence was determined through integration of clinical presentation, prior histopathologic confirmation in 2016, and comprehensive exclusion of infectious, malignant, and autoimmune mimics.

On her first presentation in 2016, empiric cefepime was started for neutropenic fever. She was put on prednisone 60 mg daily, tapered by 10 mg weekly. Fevers and arthralgia resolved within two weeks; discharged afebrile on steroid taper with rheumatology follow-up. On her 2025 readmission, following biopsy collection, prednisone 30 mg daily was initiated per rheumatology with tapering by 10 mg every 7 days. Symptoms resolved promptly and no infectious complications occurred.

This case represents an exceptionally late recurrence nine years after initial diagnosis of biopsy-supported Kikuchi–Fujimoto disease, without evidence of systemic lupus erythematosus or malignant transformation. A clinical timeline of her recurrence is presented in Supplementary Fig. 2.

## Discussion

KFD is an uncommon self-limited lymphadenitis affecting primarily young women (3,4). Its clinical resemblance to lymphoma or SLE lymphadenitis often leads to extensive and unnecessary workups. However, characteristic histology—necrotizing lymphadenitis rich in histiocytes and plasmacytoid dendritic cells—distinguishes it from other causes. Also, the absence of neutrophils in KFD makes other causes such as SLE lymphadenitis or infection less likely. The pathognomonic feature of necrosis is reported in up to 90% of KFD cohorts’ biopsies but can also be absent in some cases (12). Clinical correlation should always be established.

We present a patient with an extremely rare relapse after nine years. Most recurrences of Kikuchi–Fujimoto disease happen within the first few years, usually 1–2 years after the initial episode (13). However, longer intervals have been reported (14). A nine-year symptom-free period, as seen in our patient, is therefore unusual but not unprecedented. This case demonstrates that KFD can recur with a slightly different manifestation, in this case lymphocytic vasculitis and reactive lymphadenitis, even after a very long period of remission.

A case-based review was conducted according to CaBArET principles using PubMed/MEDLINE from 1972 through December 2025. The predefined search strategy yielded 38 records. After removal of non-English articles and reports without histopathologic confirmation, 20 studies met the inclusion criteria. One additional case was identified through reference screening, resulting in 21 total cases included in the review. The screening and data extraction process was performed independently by two authors (AA and VAF). A PRISMA-style flow diagram is provided in Supplementary Fig. 1. The following search string was applied.

(“Kikuchi-Fujimoto disease”[Title/Abstract] OR “histiocytic necrotizing lymphadenitis”[Title/Abstract]) AND (“recurrence”[Title] OR “recurrent”[Title] OR “relapse”[Title] OR “relapsing”[Title] OR “reappearance”[Title] OR “flare”[Title]) AND (“case report”[Publication Type] OR “case reports”[Title/Abstract] OR “case”[Title/Abstract] OR “patient”[Title/Abstract]).

Kikuchi–Fujimoto disease (KFD) is traditionally described as a self-limited, monophasic necrotizing lymphadenitis affecting young adults with a first manifestation in their 2nd and 3rd decade (15). The patient’s nine-year symptom-free interval followed by a new episode of febrile cervical lymphadenopathy underscores an important point: recurrence can occur even after very long latency. Synthesis of the 21 recurrent cases included in this review demonstrates that recurrence is neither rare nor uniform and may represent a distinct clinical spectrum.

Although recurrence has often been quoted at approximately 3–4% in adults, larger observational cohorts suggest higher rates. In a Taiwanese cohort of nearly 200 patients, recurrence occurred in 14.6% during follow-up, with ≥ 6-month follow-up (median interval 2 years, range 7 months to 8 years) (16). Across the cases analyzed here, the latency between episodes ranged from as little as two weeks (17) to nearly two decades (18). Earlier clinical summaries and narrative reviews gave lower figures, but those often arise from smaller samples with shorter observation windows (19). Diagnosis of recurrent KFD is complicated by overlapping features with infection and malignancy, the potential absence of classical necrosis on biopsy, and the clinician’s low index of suspicion after a long remission interval (12). Therefore, KFD recurrence may be underestimated and subject to reporting bias. Several reports documented intervals exceeding 7–10 years (9,14,19,20), indicating that recurrence is not always an early relapse phenomenon. In one patient, a 14-year interval from diagnosis to relapse has been documented (9) and ~ 8 years in a patient with other associated autoimmune features (14). Another case describes a relapse in a HTLV-I (human T lymphotropic virus type I) carrier 12 years after first diagnosis. The temporal dispersion and individual autoimmune features of patient cases suggest a long-standing immunologic vulnerability capable of reactivation under yet incompletely understood conditions. In addition, this temporal variability suggests that short-term follow-up may be insufficient and that patients, particularly those with autoimmune serologies or systemic features, may benefit from longer clinical observation. Awareness of the potential for very late recurrence is therefore essential to avoid unnecessary investigations and to ensure timely histopathologic confirmation when compatible symptoms re-emerge.

Clinically, recurrence most often mirrors the initial presentation. Fever and tender cervical lymphadenopathy remain the dominant features (16,21,22). Constitutional symptoms such as fatigue, malaise, and arthralgia are frequent, and cutaneous manifestations just as in our presented case are variably reported (22,23). Cervical lymph nodes predominate across nearly all reports. Only one describes a lymphadenopathy other than cervically (14); however, important exceptions broaden the clinical spectrum. Mesenteric and retroperitoneal lymphadenopathy during pregnancy (24), generalized nodal involvement (9,25), axillary-dominant relapse (14,20), and even subdural collections preceding abdominal recurrence (26) illustrate that relapse is not anatomically confined to the neck. While uncommon, such atypical distributions highlight the need to maintain diagnostic consideration beyond classical cervical presentations.

Laboratory findings at recurrence are remarkably consistent across cases. Leukopenia or other cytopenias are frequently observed (19,22,27), accompanied by elevated inflammatory markers including ESR and CRP (16,23). LDH elevation is common (9,22), and marked hyperferritinemia characterizes cases complicated by hemophagocytic lymphohistiocytosis (HLH) (19,23). These findings reinforce that recurrent KFD remains a systemic inflammatory process rather than a localized lymph node disorder.

Histopathologically, recurrence mostly mirrors the fundamental disease pattern. Across all included cases with biopsy confirmation, the defining features of necrotizing lymphadenitis were noted on excisional lymph node biopsy: patchy paracortical necrosis, abundant karyorrhectic debris, CD68⁺ histiocytes, CD8⁺ predominant T-cell infiltrates, and relative absence of neutrophils (9,21,22). Even in patients with multiple relapses, histologic findings remained reproducible. That histological consistency is helpful: when clinical suspicion is high but fine-needle aspiration is nondiagnostic, core, or (ideally) excisional biopsy remains decisive. Interestingly, it is our reported case that shows an altered presentation on recurrence. No obvious lymph node necrosis was noted but a skin biopsy of the patient’s rashes showed a lymphocytic vasculitic pattern. Cutaneous manifestations of KFD have widely been reported, including urticarial and maculopapular eruptions. Lymphocytic vasculitis has not been systematically characterized in association with KFD. When a vasculitic rash appears together with fever and lymphadenopathy, KFD should be considered as part of the differential diagnosis and workup. This consistency strongly supports true disease reactivation rather than transformation into an alternative inflammatory or malignant process.

A recurring theme across the dataset is the association between recurrent KFD and autoimmune phenomena. In the Taiwanese cohort, recurrence was more common among patients with positive autoimmune serologies or established autoimmune disease, particularly SLE and SjD (16). Similarly, reduced complement C4 levels and increased ANA positivity have been linked to recurrent disease (16). Individual cases demonstrate recurrence in the setting of mixed connective tissue disease (20), discoid lupus (28), Sjögren-like features (14), and evolution toward SLE-associated serology (24,29). These findings must be interpreted cautiously given the recognized clinical and immunologic overlap between KFD and systemic autoimmune disorders (30). While causality cannot be established from case-based evidence, these observations suggest that, in a subset of patients, recurrent KFD may lie along a broader autoimmune continuum rather than representing an entirely isolated entity.

Mechanistic insight remains limited but intriguing. One relapsing case demonstrated marked elevations of IL-6, IL-7, IL-8, MCP-1, and TNF-α during recurrence, all of which declined with corticosteroid therapy (31). This cytokine profile suggests that recurrence reflects reactivation of a dysregulated immune process. Severe immune amplification is illustrated by cases progressing to HLH (19,23), placing recurrent KFD within a broader spectrum of hyperinflammatory syndromes. Emerging work implicates exaggerated cytotoxic T-cell activity and interferon-driven pathways, further aligning recurrent KFD with immune dysregulation rather than transient infection. Histopathological studies beyond our review of recurrent cases describe a predominance of CD8⁺ T cells accompanied by extensive apoptosis within necrotic foci (32). More recent work has drawn attention to abnormalities in innate immune pathways, including increased perforin-2 expression and activation of type I interferon signaling (33,34). Sustained or dysregulated interferon responses, particularly those driven by plasmacytoid dendritic cells, may lower the threshold for renewed immune activation and may explain why some patients experience delayed relapse.

Treatment responses further support an immune-mediated mechanism. Many recurrences resolve with supportive care, nonsteroidal anti-inflammatory drugs (NSAID), or short corticosteroid courses (18,35). However, steroid dependency and relapse during tapering were repeatedly described (22,36). In refractory cases, hydroxychloroquine was used as an add-on to prednisolone (PSL) (22) and even IL-1 inhibition with anakinra (25) has been used to achieve sustainable remission, particularly when autoimmune overlap was suspected. Rituximab has been used in selected cases with suspected immune overlap (26). Although evidence remains limited to case reports, these observations suggest that targeted immunomodulation may be justified in selected recurrent or steroid-dependent patients. Further research is needed to clarify this approach. Treatment should be individualized and patient-specific in recurrent cases taking into consideration severity of symptoms.

Age distribution in recurrence appears broader than that described for primary disease. While most primary manifestation cases occur in young adults, recurrence has been documented in adolescents (37) and especially in patients over 50 years of age (35), indicating that relapse is not restricted to the typical demographic profile of first presentation. Beyond our review, KFD has a reported recurrence rate in pediatric populations of up to 42.4% (38).

Ethnically, the majority of recurrence reports originate from East Asian populations, consistent with the higher overall incidence of KFD in this region (16,22,27). Nevertheless, recurrent disease has been reported across diverse ethnic backgrounds including African, European, Middle Eastern, and Hispanic patients (14,25,35,36). Thus, while geographic predominance likely reflects biological and reporting factors, recurrence is not ethnically confined and can be seen across all patient ethnicities. The marked predominance of Asian patients across published KFD recurrence cases likely reflects a multifactorial phenomenon rather than a purely geographic artifact. Genetic predisposition, particularly the higher frequency of HLA class II alleles such as HLA-DPA1 and DPB1 variants described in East-Asian have been proposed as possible contributors (34). Environmental or infectious co-triggers, including region-specific viral exposures, could further amplify disease expression in genetically predisposed hosts. Nevertheless, reporting and detection bias remain possible: heightened clinical awareness and routine lymph node biopsies in East Asia may lead to overrepresentation in literature (34).Broader, multiethnic cohort studies are needed to disentangle these factors and determine whether ethnicity itself confers a true biological risk for Kikuchi–Fujimoto disease recurrence.

Taken together, the collective evidence from these 21 recurrent cases suggests that recurrent KFD represents a heterogeneous but biologically coherent spectrum of immune dysregulation. The wide latency distribution, consistent histopathology, enrichment of autoimmune features, occasional progression to HLH, and steroid responsiveness all point toward a relapsing immune-mediated process rather than isolated episodic lymphadenitis. Further multicenter, longitudinal studies are needed to clarify predictors of recurrence, define the role of autoimmune predisposition, and determine whether early immunomodulatory intervention alters the natural course in high-risk individuals.

## Supplementary Information

Below is the link to the electronic supplementary material.

ESM 1 PNG (829 KB)

ESM 2 PNG (83.8 KB)

ESM 3 PNG (577 KB)
