# Supplementary figures and images for: Late recurrence of Kikuchi–Fujimoto disease nine years after initial diagnosis: a case-based review
Source: Clin Rheumatol. 2026 Apr 30;45(7):4647–54. doi: 10.1007/s10067-026-08134-7 (PMC13341919; doi:10.1007/s10067-026-08134-7)

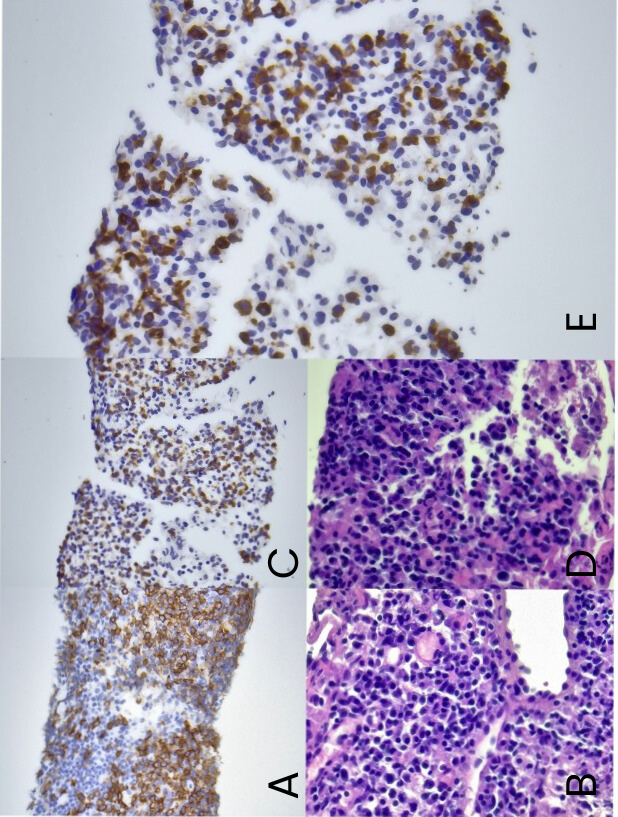

Supplement: Supplementary file 1 — PNG (829 KB) [file 10067_2026_8134_MOESM1_ESM.png]

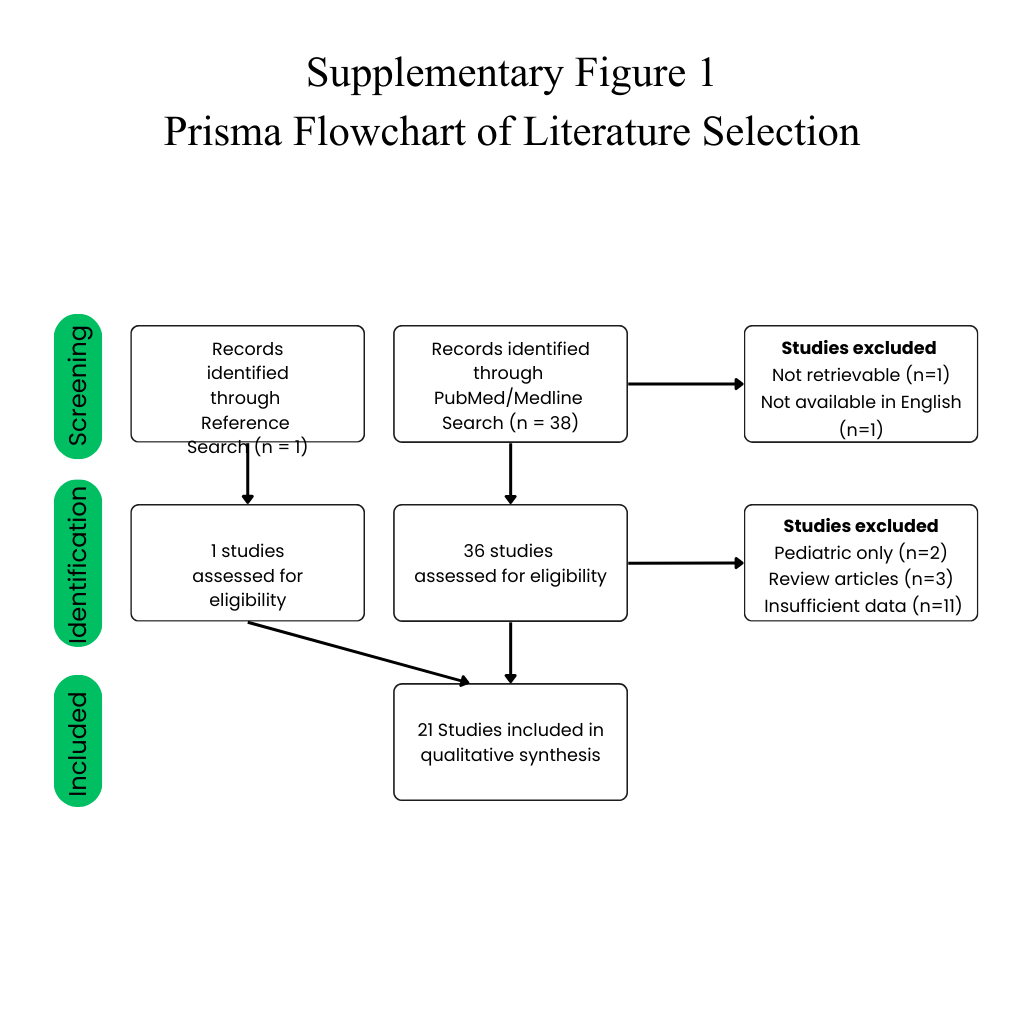

Supplement: Supplementary file 2 — PNG (83.8 KB) [file 10067_2026_8134_MOESM2_ESM.png]

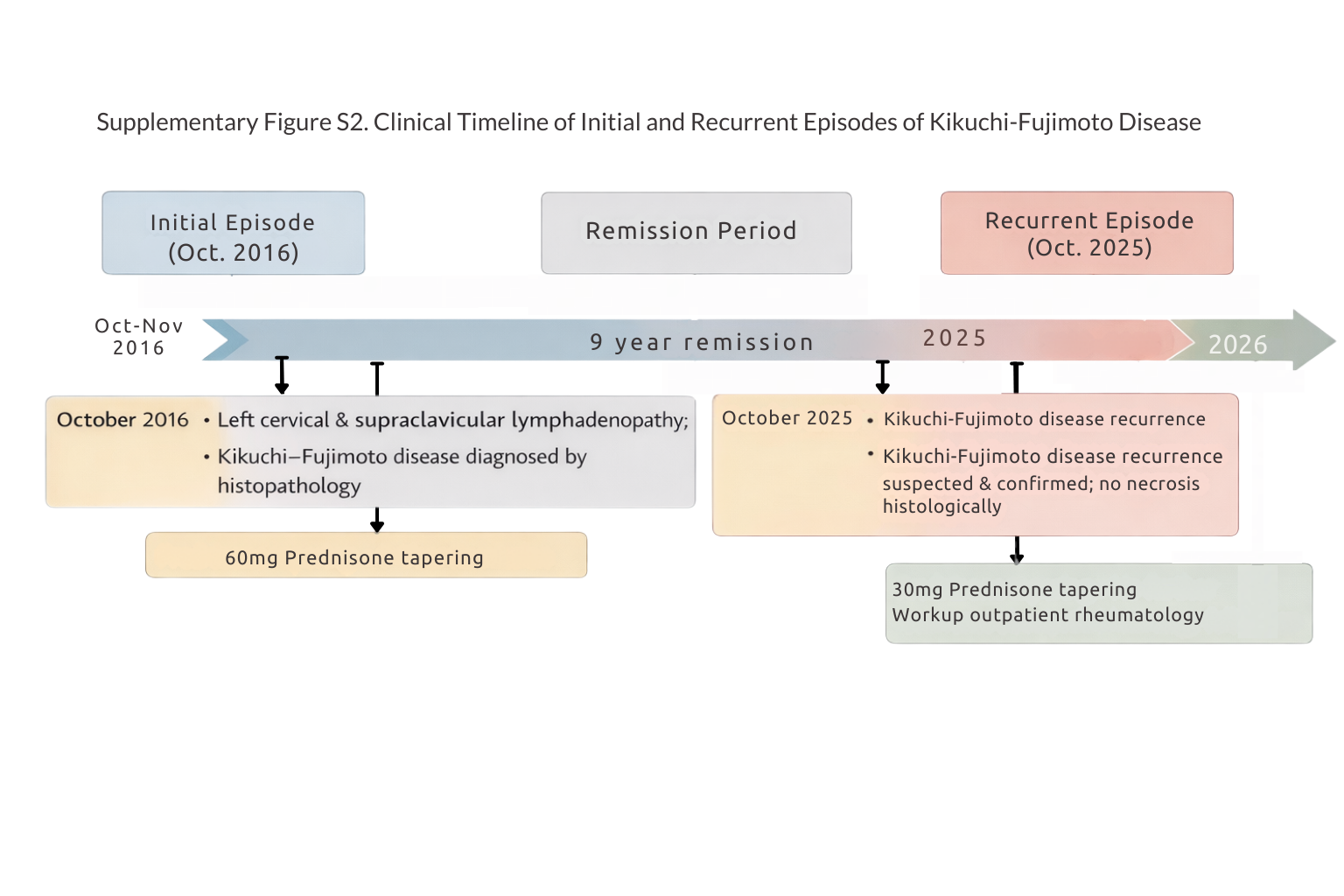

Supplement: Supplementary file 3 — PNG (577 KB) [file 10067_2026_8134_MOESM3_ESM.png]
